# Mortality in patients with interstitial lung diseases hospitalized by severe or critical COVID-19

**DOI:** 10.1186/s12890-023-02697-w

**Published:** 2023-10-13

**Authors:** Ana Karem S. Pruneda, José Omar Barreto-Rodríguez, Moises Selman, Fortunato Juárez-Hernández, Ivette Buendía-Roldán

**Affiliations:** Instituto Nacional de Enfermedades Respiratorias, Ismael Cosío Villegas, Calzada de Tlalpan 4502, Col. Sección XVI, 14080 Mexico, Tlalpan Mexico

**Keywords:** Interstitial lung diseases, Severe COVID-19, Critical COVID-19 mortality

## Abstract

**Background:**

Since the first case of severe COVID-19, its effect on patients with previous interstitial lung disease (ILD) has been uncertain. We aimed to describe baseline clinical characteristics in ILD patients hospitalized by critical COVID and compare mortality during hospitalization.

**Methods:**

We studied patients with ILD with COVID-19 and a control group matched by age, 1:2 ratio with COVID-19 without previous lung disease. On admission, laboratory tests and sociodemographic variables were evaluated. We evaluated patients critically ill and compared baseline characteristics and mortality in each group. Additionally, we performed a sub-analysis of ILD patients who died versus survivors.

**Results:**

Forty-one patients and 82 controls were analyzed. In the group of ILD with COVID-19 there was a predominance of women (65 versus 33%: *p* < 0.001); lower leukocytes (9 ± 6 versus 11 ± 7, *p* = 0.01) and neutrophils (8 ± 5 versus 10 ± 6, *p* = 0.02). The most common ILD was secondary to autoimmune diseases. Patients with ILD and critical COVID-19 showed a significantly higher mortality compared with those without previous ILD (63 versus 33%, *p* = 0.007). Patients who died in this group had higher BMI (28 ± 6 versus 25 ± 4 kg/m^2^, *p* = 0.05), less extended hospital stay (20 ± 17 versus 36 ± 27 days, *p* = 0.01), and fewer days of evolution (9 ± 7 versus 16 ± 16, *p* = 0.05).

**Conclusions:**

We found higher mortality in patients with ILD with critical COVID-19. Higher BMI and comorbidities were present in the non-survivors.

## Background

Since 2020, the world has been facing a pandemic caused by the SARS-CoV2 virus, known as COVID-19, [1, 2] which presents heterogeneity in its presentation, initially with severe or critical cases and post-vaccine mild to moderate [3].

In some studies, has been reported that patients with comorbidities had twelve times more risk of death secondary to severe COVID-19 and six times more risk of requiring hospitalization [4].

Yang and cols. defined critically ill patients as those who required admission to the intensive care unit with mechanical ventilation or a requirement of FiO2 of at least 60% [5].

In this context, the impact of severe COVID-19 on patients with interstitial lung diseases (ILD) was an imminent concern; some authors developed studies to describe mortality in this group of patients: A multicentric European study reported a mortality of 49% versus 35% in patients without lung diseases (*p* = 0.013), 84% of the patients in the group ILD and 79% of the control group received supplementary oxygen through high-flow oxygen devices. The group that did not receive high-flow nasal oxygen devices had better survival than those that received them (93% versus 75%) [6]. A retrospective study in a tertiary care hospital in India with 30 ILD patients obtained mortality of 35.7% [7].

In Mexico, the Instituto Nacional de Enfermedades Respiratorias (INER) is a reference center for interstitial lung diseases; during the pandemic was a center of exclusive attention for severe and critically severe COVID-19, so this study aimed to describe baseline clinical characteristics and the ILD more frequently presented in patients hospitalized by severe or critical COVID and compare mortality during hospitalization.

## Materials and methods

### Study population

We included all patients admitted to the hospital with a confirmed diagnosis of COVID-19, defined with a positive PCR swab test to SARS-CoV2, with a severe or critical presentation, and who had an ILD diagnosis previously in our Institute from March 2020 to January 2022. The control group was randomized among patients hospitalized without previous respiratory diseases but with confirmed severe or critical COVID-19 matched by age with a 1:2 ratio.

We defined severe or critical COVID-19 as reported in severe COVID-19 Treatment Guidelines:

*Severe Illness: Individuals who have SpO2 < 94% on room air at sea level, the ratio of arterial partial pressure of oxygen to fraction of inspired oxygen (PaO2/FiO2) < 300 mm Hg, respiratory rate > 30 breaths/min, infiltrates in computed tomography > 50% [8].
*Critical Illness: Individuals who have respiratory failure, septic shock, and multiple organ dysfunction [8].

At admission, all the patients underwent computed tomography, laboratory tests with inflammatory biomarkers, and a complete clinical chart review including comorbidities, smoking history, and physical exploration. Patients with incomplete medical records or mild or severe COVID-19 disease that did not require hospitalization were excluded. All the information was obtained from electronic medical records.

We classified patients into four groups: patients with ILD with severe or critically ill and patients without previous respiratory disease with severe or critically ill (controls); we compared baseline characteristics and mortality in each group. Finally, we performed a sub-analysis of the patients with previous ILD who died versus the survivors.

#### Ethics and consent

This study was approved by the Investigation and Bioethics Committee of the National Institute of Respiratory Diseases (C20-21). The results obtained guaranteed the protection of individual rights and maintained confidentiality; we obtained informed consent from all patients; in cases where the patient was sedated, authorization was requested from the responsible family member.

#### Statistical analysis

We performed a normality test with the Kolmogorov–Smirnov test. The descriptive data were presented as frequency, percentages, and mean and standard deviation. Comparison between groups was performed with Fisher- exact test for qualitative variables and U-Mann–Whitney for quantitative variables. We used the statistical program GraphPad Prism V8. *p*-values < 0.05 were considered statistically significant.

## Results

We studied 41 ILD with COVID-19 patients and 82 patients with COVID-19 without chronic respiratory diseases. The mean age in both groups was over 60 years old. We found a difference in gender in the ILD group, with a predominance of women (65% versus 33%, *p* = 0.0005). We did not find any difference in comorbidities, basal oxygen saturation, length of hospital stays, or evolution days. Leukocytes were significantly higher in the control group than in the ILD group (11 ± 7 versus 9 ± 6, *p* = 0.01), caused by higher neutrophils (10 ± 6 versus 8 ± 5, *p* = 0.02). CRP and procalcitonin levels were more elevated in the control group compared to ILD patients without a statistical difference, as shown in Table 1.

**Table 1:** Demographic data and clinical characteristics

Variable	ILD with COVID-19 (*n* = 41)	COVID-19 (*n* = 82)	*P*-value
Age, years, IQR	65 (57–75)	64 (59–72)	0.8
Gender, women (%)	27 (65)	27 (33)	0.0005
BMI (kg/m2), IQR	27	28 (25—32)	0.3
Type 2 diabetes, (%)	8 (20)	25 (30)	0.13
Systemic high blood pressure (%)	14 (34)	32 (39)	0.3
Smoking status (%)	14 (34)	24 (29)	0.3
Complete vaccination (%)	6 (14)	7 (9)	0.2
Oxygen saturation at rest, IQR	71 (62–85)	65 (54—80)	0.13
Days of evolution, IQR	10 (6–15)	10 (7—13)	0.7
Leukocytes absolute number (10^3/mm^3^), IQR	9 (6–12)	11 (8—14)	0.01
Neutrophiles absolute number (10^3/mm^3^), IQR	8 (5–10)	10 (6—12)	0.02
Lymphocytes absolute number (10^3/mm^3^), IQR	0.8 (0.3–1)	0.8 (0.5—1)	0.1
CRP (mg/dL), IQR	15 (8–22)	19 (11—25)	0.06
Procalcitonin (ng/dL), IQR	0.6 (0.06–0.4)	0.7 (0.1—0.5)	0.09
Ferritin (ng/dL), IQR	1246 (445–1687)	1592 (588—2202)	0.1
Fibrinogen (mg/dL), IQR	639 (472–750)	698 (600–750)	0.1
D-Dimer (μg/mL), IQR	3.6 (0.6–3.7)	4 (0.7–3)	0.9

*BMI* Body mass index, *CRP* C-reactive protein, *ILD* Interstitial lung diseases, *IQR* Interquartile range

### Oxygen requirements and hospital outcomes

During their hospital stay, 24 ILD patients (57%) and 72 patients in the control group (88%) required invasive mechanical ventilation (IMV), 11 patients in the ILD group (27%) and 8 in the control group (10%) required high flow nasal cannula and 6 ILD patients (14%) and 2 in the control group (2%) required only low flow oxygen devices, without significant differences in either group. In the ILD group, 45% died during their hospital stay versus 30% in the control group Table 2.

**Table 2:** Outcomes and oxygen requirements

Variable	ILD with COVID-19 (*n* = 41)	COVID-19 (*n* = 82)	*P*-value
IMV, (%)	24 (57)	72 (88)	0.0003
HFNC, (%)	11 (27)	8 (10)	0.01
SFC/SM, (%)	6 (14)	2 (2)	0.01
Death, (%)	19 (45)	25 (30)	0.06

*IMV* invasive mechanical ventilation, *HFNC* high Flow nasal cannula, *SFC* simple Flow cannula, *SM* simple mask, *ILD* interstitial lung disease

**Fig. 1:**
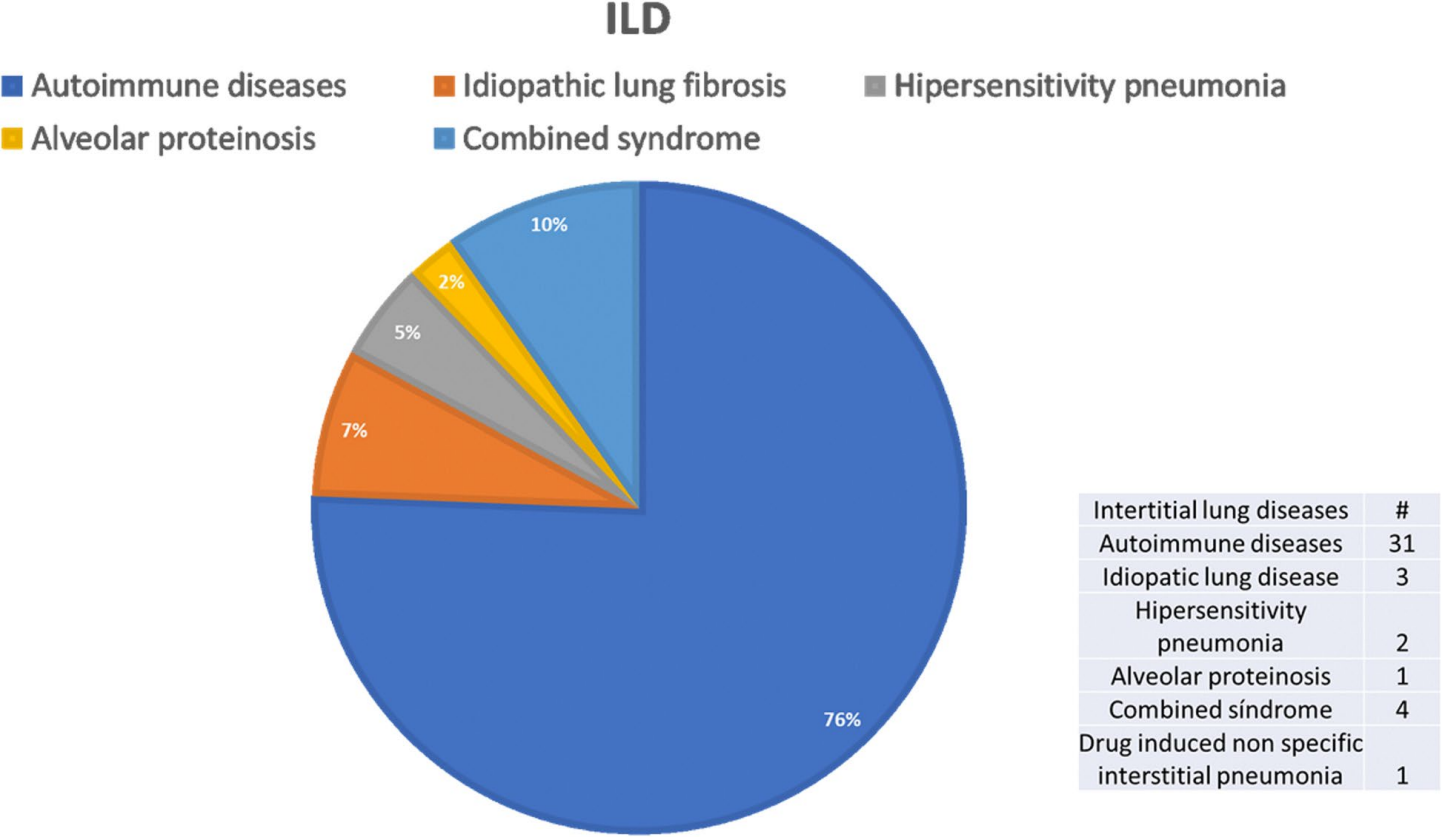
This graphic showed the frequency of the different previous diagnoses in the interstitial lung diseases group. The most frequent was secondary to autoimmune diseases

Furthermore, we obtained the type of ILD diagnosis in the patients with COVID-19: 31 patients with autoimmune diseases related to interstitial lung disease, three had idiopathic pulmonary fibrosis, two had fibrotic hypersensitivity pneumonia, one alveolar proteinosis, four patients presented emphysema and lung fibrosis (combined syndrome) and one drug-induced non-specific interstitial pneumonia Fig. 1.

### Differences between groups of Critical and Severe COVID-19

When we analyzed patients according to their clinical severity into severe COVID-19 and critical COVID-19, we detected 27 patients with ILD in the critical group and 72 patients in the control group, while 14 patients in the ILD group were classified as severe COVID-19 group compared to only ten patients from the control group, so we only compared the critical severity and significant differences were observed in gender, with a predominance of women (63% versus 31%, *p* = 0.003). The ILD with critical COVID-19 group had significantly higher mortality than the control group (63% vs. 33%, *p* = 0.007) Table 3.

**Table 3:** Demographic data and clinical characteristics of patients with critical COVID-19

Variable	Critical COVID-19 with ILD (*n* = 27)	Critical COVID-19 (*n* = 72)	*P*-value
Age (years), IQR	62 (55–69)	65 (60–71)	0.3
Gender (Women), (%)	17 (63)	22 (31)	0.003
BMI (kg/m2), IQR	27 (25–29)	29 (25–32)	0.4
Type 2 diabetes, (%)	6 (22)	19 (26)	0.4
Systemic high blood pressure (%)	8 (30)	28 (39)	0.2
Complete vaccination (%)	5 (19)	6 (8)	0.1
Oxygen saturation (%) IQR	70 (61–82)	64 (52–78)	0.2
Days of evolution (#) IQR	11 (7–16)	10 (7–13)	0.7
Leukocytes absolute number (10^3/mm^3^), IQR	10 (7–13)	12 (8–14)	0.1
Neutrophiles absolute number (10^3/mm^3^), IQR	9 (6–12)	10 (6–13)	0.3
Lymphocytes absolute number (10^3/mm^3^), IQR	0.7 (0.3–1)	0.8 (0.5–1)	0.07
CRP (mg/dL), IQR	18 (11–24)	19 (11–26)	0.5
Procalcitonin (ng/mL), IQR	0.8 (0.07–0.6)	0.6 (0.1–0.6)	0.6
Ferritin (ng/mL), IQR	1325 (449–1693)	1628 (669–2190)	0.2
Fibrinogen (mg/dL), IQR	705 (677–779)	713 (624–776)	0.7
D-Dimer (μg/dL), IQR	5 (0.8–4.8)	4.1 (0.7–3.5)	0.3
Dead (%)	17 (63)	24 (33)	0.007

*BMI* body mass index, *CRP* C-reactive protein, *ILD* interstitial lung diseases, *IQR* interquartile range

We performed a sub-analysis to compare the clinical, sociodemographic, and laboratory findings among the patients with ILD who died vs. the survivors. The patients who died had a higher BMI (28 ± 5 vs. 26 ± 5, *p* = 0.02) without other differences in the other variables, including vaccination status Table 4.

**Table 4:** Demographic data and clinical characteristics of dead and survivors ILD patients with COVID-19

Variable	ILD with COVID-19 dead (*n* = 19)	ILD with COVID-19 survivors (*n* = 22)	*P*-value
Gender (women), (%)	14 (73)	13 (59)	0.2
BMI (kg/m2), IQR	28 (26–31)	26 (23–28)	0.02
Type 2 diabetes, (%)	5 (26)	3 (13)	0.2
Systemic high blood pressure (%)	8 (42)	7 (30)	0.3

Finally, in the critical ILD with COVID-19 group were 17 non-survivors and ten survivors. The BMI was significantly higher in the non-survivors’ group (28 ± 6 versus 25 ± 4, *p* = 0.05), the length of stay and days of evolution was significantly longer in the survivors' group (20 ± 11 versus 36 ± 24, *p* = 0.01; 9 ± 7 versus 16 ± 16, *p* = 0.05, respectively) Table 5.

**Table 5:** Demographic data and clinical characteristics of dead versus survivors ILD patients with critical COVID-19

Variable	ILD with critical COVID-19 dead (*n* = 17)	ILD with critical COVID-19 survivors (*n* = 10)	*P*-value
Gender women (%)	12 (70)	5 (50)	0.3
BMI (kg/m2), IQR	28 (25–31)	25 (23–27)	0.2
Diabetes mellitus (%)	4 (24)	2 (20)	0.05
Systemic high blood pressure (%)	7 (41)	1 (10)	0.6
Smoking (%)	3 (18)	3 (30)	0.09
Complete vaccination (%)	2 (12)	3 (30)	0.3
Days of hospital stay, IQR	20 (12–29)	36 (18–46)	0.2
Days of evolution, IQR	9 (5–12)	16 (7–23)	0.01
PaO2/FiO2, IQR	140 (93–172)	176 (130–242)	0.05
Leukocytes absolute number(10^3/mm^3^), IQR	9 (8–11)	12 (6–14)	0.2
Neutrophiles absolute number(10^3/mm^3^), IQR	8 (6–9)	10 (6–13)	0.2
Lymphocytes absolute number(10^3/mm^3^), IQR	0.7 (0.3–0.9)	0.6 (0.3–1)	0.2
CRP (mg/dL), IQR	17 (12–23)	19 (10–25)	0.9
Procalcitonin (ng/mL), IQR	1 (0.1–1.3)	0.4 (0.04–0.5)	0.6
Ferritin (ng/mL), IQR	1207 (449–1688)	1561 (429–2085)	0.2
Fibrinogen (mg/dL), IQR	681 (656–771)	736 (686–956)	0.8
D-Dimer (μg/dL), IQR	3 (1–5)	7 (0.4–4)	0.6

*BMI* body mass index, *SD* standard deviation, *DD* D dimer, *CRP* C-reactive protein, *ILD* interstitial lung diseases

## Discussion

In recent years, COVID-19 in patients with ILD has been reported to be associated with poor outcomes, but the reason is unclear [9, 10]. Lee et al. studied a national cohort of patients with severe COVID-19 (*n* = 8070) and controls from the same geography without a diagnosis of severe COVID-19 (*n* = 121,050). They evaluated the number of patients with ILD in both groups and found that patients with ILD had a more significant probability of having severe COVID-19, in comparison with the rest of the population (0.8 versus 0.4% *p* =  < 0.001), of presenting a more severe presentation of the disease (OR 2.23, IC 95% 1.24–4.01) and of dying (13.4% vs. 2.8%, *p* =  < 0.001) [9]. Immediately, ILD centers performed different preventive interventions to avoid severe forms of pulmonary infection during the pandemic. For example, in a Belgian center of their ILD cohort with 400 patients, only 4 had severe COVID-19 that required hospitalization, and 33 cases were suggestive to SARS-CoV2 infection [11].

In our cohort of ILD with COVID-19, we found a predominance of women; this finding may be because most of the patients with ILD were secondary to autoimmune diseases, compared to the study by Drake and Cols [6] who reported that the most frequent ILD was IPF (42% of the cases), while Dutt et al., in their study, reported that the most frequent was NSIP with 32% [7]. Also, Gallay et al. reported that patients with IPF had a higher risk of death secondary to severe COVID-19 than other interstitial lung diseases (35% versus 19%, *p* = 0.04) [12]. Also, Naqvi and Cols. in a matched multicentre research network analysis, reported that 44% of patients with COVID-19 and comorbid IPF required hospitalization and in this group mortality rate of one in three was observed [13].

The age of our population was like that published in the other studies. Also, we found lower levels of leukocytes and neutrophils in the ILD group and a trend in inflammatory markers such as lower CRP and procalcitonin; these results could be influenced by the chronic use of systemic steroids in the ILD group. However, similar results had been reported with a higher number of neutrophils, monocytes, and D-Dimer in COVID-19 with ILD compared to non-ILD COVID-19 patients [14].

We classified the patients according to their severity according to COVID-19 guidelines [8], and our results agree with higher mortality in patients with ILD with critical COVID-19 (63% vs. 33%, *p* = 0.007), as was expected. We consider that this increase in mortality is influenced by the previous structural alterations of the patients that make them a complex group to ventilate, challenging to maintain alveolar protection goals, with a low functional reserve, and difficult to extubate. It had previously been reported a greater than four-fold increased adjusted odds of death and required intensive care unit (ICU) level in patients with ILD who contracted COVID-19 [15], also Cilly and Cols., reported in a Turkey cohort, that hospitalization rate was 84%. ICU admission was 21% in fibrotic idiopathic ILD [16].

We did not find a difference in mortality in the severe COVID-19 group between patients with ILD and those without known lung diseases, unlike what was reported in other studies, but patients with Sjogren’s syndrome were reported with a reduced SARS-CoV2 mortality by Zhao J et al. [17], following our results. We believe this could be due to the small number of patients with severe COVID-19; it is one of the most significant limitations because it is a single reference center study.

Finally, we decided to compare the differences between the patients who died in the ILD group versus the survivors, and we found differences in BMI, which was higher in the group of patients who died; this result is consistent with other studies on the influence of BMI on mortality [4]. The non-survivors group showed a trend in present high blood pressure compared to survivors, which has also been described as a risk factor for poor prognosis. Also, the difference in the number of days of evolution in the non-survivor’s group (12 days) related to the significant inflammation is interesting.

The strength of our study is that we analyzed ILD with COVID-19 versus COVID-19 without previous lung disease and the sub-analysis between dead and survivors. In the future, it is essential to follow up with the group of patients with ILD with severe COVID-19 to determine if this infection is a risk factor for developing progressive pulmonary fibrosis.

## Conclusions

We found higher mortality in the group of ILD patients with critical COVID-19. Higher BMI and comorbidities were present in the non-survivors.

## Data Availability

All data generated or analysed during this study are included in this published article.

## Abbreviations

ILD: Interstitial lung disease
COVID-19: Coronavirus disease -19
BMI: Body mass index
CRP: C-reactive protein
IQR: Interquartile range
IMV: Invasive mechanical ventilation
HFNC: High flow nasal cannula
SFC: Simple flow cannula
SM: Simple mask
SpO2: Oxygen saturation
DD: D dimer
